# Berberine and its nanoformulations and extracts: potential strategies and future perspectives against multi-drug resistant bacterial infections

**DOI:** 10.3389/fmicb.2025.1643409

**Published:** 2025-09-02

**Authors:** Xue Yang, Yanfen Wang, Ling Li, Daiyan Tang, Zhong Yan, MingYan Li, Jiayi Jiang, Dongming Bi

**Affiliations:** ^1^Department of Laboratory Medicine, Hospital of Chengdu University of Traditional Chinese Medicine, Chengdu, Sichuan, China; ^2^Department of Laboratory Medicine, Deyang Hospital Affiliated Hospital of Chengdu University of Traditional Chinese Medicine, Deyang, Sichuan, China; ^3^Department of Clinical Laboratory, Ya’an People’s Hospital, Yaan, Sichuan, China; ^4^College of Medical Technology, Chengdu University of Traditional Chinese Medicine, Chengdu, China; ^5^Department of Nuclear Medicine, Ya’an People’s Hospital, Yaan, Sichuan, China

**Keywords:** berberine, bacterial, antimicrobial resistance, natural products, antimicrobial agents

## Abstract

The increasing prevalence of antimicrobial resistance (AMR) has led to the gradual decline in the effectiveness of existing antibiotics, posing a significant threat to global health. Many phytochemicals have antimicrobial activity, but few have been developed for clinical use. Berberine, an alkaloid found in various medicinal plants, has been recognized as a promising strategy to combat AMR due to its notable antimicrobial activity and role in reversing resistance. Here, we present a systematic, comprehensive and objective overview of the antimicrobial activity, mechanism of action, and limitations of berberine. Additionally, we discuss the antimicrobial efficacy of berberine extracts and nanoformulations. Berberine demonstrates broad-spectrum antimicrobial activity by inhibiting FtsZ, disrupting cell membranes and cell walls, and interfering with DNA and RNA synthesis. However, due to its low bioavailability and lack of systematic *in vivo* validation, the efficacy of berberine as a standalone treatment for bacterial infections requires further investigation. Nevertheless, it can serve as an antibiotic adjuvant to enhance the efficacy of conventional antibiotics and reverse AMR. Moreover, the excellent antimicrobial effects exhibited by berberine extracts and nanoformulations may overcome these limitations, representing potential future applications of berberine. In conclusion, berberine has great potential as an antimicrobial agent and antibiotic adjuvant in combating AMR, but systematic and comprehensive *in vivo* and clinical trials are still needed to evaluate the therapeutic efficacy of berberine and optimize its use.

## Graphical Abstract

Antibacterial mechanisms of berberine *in vitro* and *in vivo* and its future applications. 
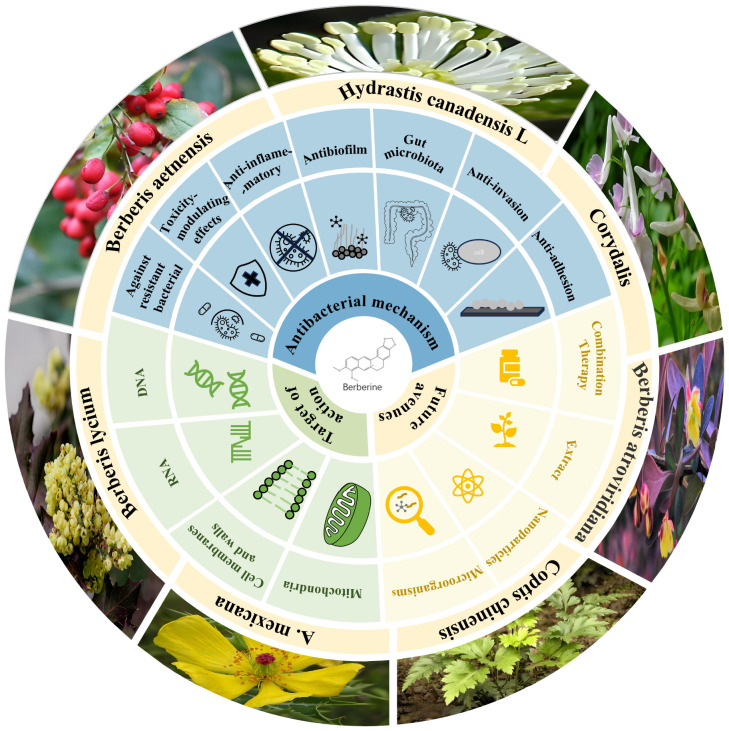

## 1 Introduction

In recent decades, the rise of antimicrobial resistance (AMR) has elevated bacterial infections to one of the most pressing global public health threats (Larkin, 2023). Pathogenic microorganisms have developed various resistance mechanisms through continuous adaptation and evolution, such as the production of inactivating enzymes, reduced membrane permeability, and antibiotic efflux pumps, which have reduced the available options and clinical efficacy of antibiotics, leading to alarming increases in mortality (Guedes et al., 2024). In 2021, approximately 4.71 million deaths globally were associated with AMR, with 1.14 million directly attributed to AMR. Projections suggest that by 2050, AMR could result in 8.22 million related deaths annually, including 1.91 million directly caused by resistant infections (Kariuki, 2024). This alarming trend is fueled by the overuse and misuse of antibiotics in healthcare and agriculture, a lack of new antimicrobial agents, and inadequate infection control strategies (Hays et al., 2022; Caioni et al., 2024; Lewnard et al., 2024). Importantly, the declining cost-effectiveness of developing new antibiotics, combined with the lack of direct inhibitory effects of resistance mechanism inhibitors on bacterial cells, has resulted in a severe imbalance between the urgent need for antibiotics and the current pace of their development (Seukep et al., 2020; Cook and Wright, 2022). Therefore, there is an urgent need to develop broad-spectrum antibiotics that not only exhibit direct bactericidal activity but also effectively counter AMR.

**FIGURE 1:**
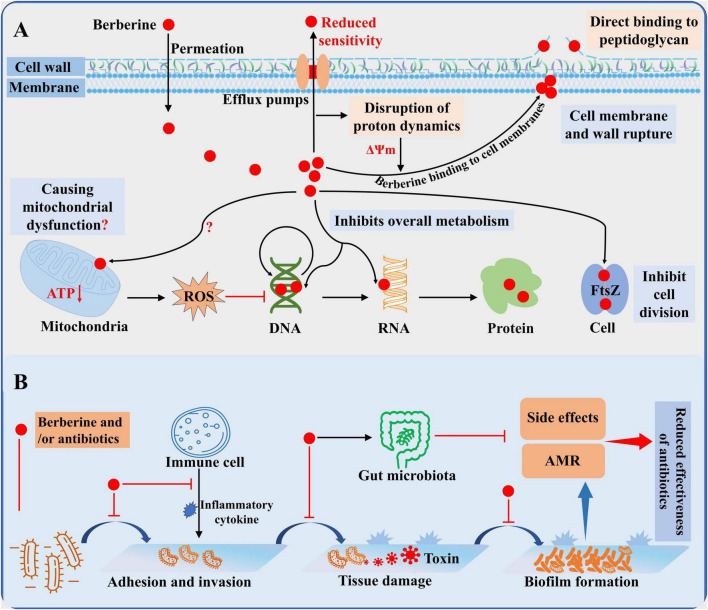
General overview of the antibacterial activity of berberine. **(A)** Antibacterial mechanism of berberine. **(B)** Mode of action of berberine alone or in combination *in vivo*.

Today, pharmacologically active plants continue to serve as the primary pharmacopeia in many developing countries, with their clinical efficacy proven through centuries of traditional medicine (Porras et al., 2021). Regrettably, between 1981 and 2019, 50% of the 162 antimicrobials approved by the U.S. Food and Drug Administration were derived from microbial natural products and their derivatives, rather than from plant sources (Porras et al., 2021). However, many excellent recent reviews describe the great potential of plant natural products such as phenolic derivatives, terpenoids, and alkaloids as antimicrobial agents (Newman and Cragg, 2020; Herman and Herman, 2023; Lu et al., 2024). Among them, berberine is considered one of the most promising candidates for antimicrobial drug development. Found in medicinal plants such as *Hydrastis canadensis*, *Berberis aristata*, *Coptis rhizome*, *Coptis japonica*, and *Phellodendron amurense*, berberine has a long history of therapeutic use worldwide (Gasmi et al., 2024). It exhibits broad-spectrum antiviral and antifungal activity both *in vitro* and *in vivo* and has been shown to act as an antibiotic adjuvant, reversing fungal and bacterial resistance (Warowicka et al., 2020; Zhou H. et al., 2023; Ding et al., 2024). In addition, berberine exhibits a range of other pharmacological effects, including anti-tumor, anti-inflammatory, antimicrobial, and cardiovascular protective properties (Patel, 2021). These attributes enhance its economic viability and clinical application. More importantly, berberine’s low cost, availability, and accessibility offer a practical and feasible strategy for managing antibiotic resistance, particularly in developing countries. Against this backdrop, we provide a comprehensive and systematic review of berberine’s antimicrobial activity and mechanisms, as well as its limitations, with a focus on its effects on a range of pathogenic bacteria over the past two decades (Figure 1). Furthermore, we describe the antimicrobial properties of berberine-containing natural extracts and nanoformulations, exploring potential pathways for its future clinical applications. By addressing the global challenge of bacterial infections, this review aims to provide a theoretical foundation for the further development of berberine and offer practical solutions for managing global AMR.

## 2 Literature search strategy

A comprehensive literature search was conducted in three major databases: PubMed, Google Scholar, and Web of Science. The search was restricted to English-language publications from 2000 to 2025. Keywords used included “Berberine,” “Bacteria,” “Antibacterial activity,” “Antibacterial mechanism,” “*In vivo*,” “*In vitro*,” “Nanoparticles,” “Extracts,” and various combinations of these keywords. The initial search results were imported into EndNote software for reference management and removal of duplicates. Titles, abstracts, and full texts of the retrieved articles were carefully screened for relevance. Studies were included if they investigated the antibacterial effects and mechanisms of berberine, its nanoformulations, or natural extracts, either *in vitro* or *in vivo*, and provided the source of berberine whenever available. Studies not involving bacterial pathogens, relevant infection models, or those unrelated to berberine-based interventions were excluded from the analysis.

## 3 Antibacterial activities of berberine

### 3.1 Antibacterial properties of berberine against pathogenic bacteria *in vitro*

Berberine exhibits broad-spectrum antimicrobial activity (Table 1) and demonstrates moderate efficacy against various pathogens, including World Health Organization priority pathogens such as *Acinetobacter baumannii*, *Pseudomonas aeruginosa*, *Enterococcus faecalis*, and *Staphylococcus aureus*. It also inhibits the growth and proliferation of *Prevotella bryantii*, *Bacteroides fragilis*, *Acetoanaerobium sticklandii*, and *Porphyromonas gingivalis* (Lakes et al., 2020; Okuda et al., 2023). Meanwhile, berberine inhibits spore growth of *C. difficile* and *Bacillus cereus*, thereby reducing the potential harm caused by spore germination (Wang et al., 2016). Moreover, antimicrobial effects of berberine are dose- and time-dependent; for instance, in *Escherichia coli* and methicillin-resistant *S. aureus* (*MRSA*), their survival rates gradually decrease with increasing concentrations of berberine or extended incubation times (Li et al., 2018c; Zhou F. F. et al., 2023). Unlike bacteriostatic agents such as chloramphenicol and clindamycin, berberine exerts bactericidal activity against various pathogens, including *MRSA*, *Staphylococcus epidermidis*, *C. difficile*, and *Salmonella typhimurium*, though the effective concentrations are substantially higher than their minimum inhibitory concentrations (MICs) (Wang et al., 2009b; Zhang et al., 2013; Peng et al., 2015; Wultańska et al., 2020). For example, the minimum bactericidal concentration for *MRSA* is 2,560 μg/mL, which is 25 times its MIC (Qiu and Xu, 2024). However, the MIC range of berberine against bacteria varies widely, from 0.78 μg/mL against *Streptococcus agalactiae* to as high as 100,000 μg/mL against *Helicobacter pylori* (Huang et al., 2015; Peng et al., 2015). Even for the same pathogen, this variability can be significant. For example, Li et al. (2018a) reported the MIC of berberine against *H. pylori* to be 25,000–100,000 μg/mL, while Huang et al. (2015) reported it to be 50 μg/mL. These discrepancies may be attributed to differences in their antimicrobial susceptibility testing methods (Columbia blood agar with agar dilution vs. Brucella broth with broth dilution). The antifungal activity of berberine is also influenced by the culture medium (Ding et al., 2024). Indeed, prolonged exposure to berberine led to increased energy demands in *E. coli*, and the amino acid maintenance strategy shifted from transport to synthesis (Budeyri Gokgoz et al., 2017). Therefore, it can be inferred that berberine’s antimicrobial activity is susceptible to the influence of nutritional substrates, which is also related to its antimicrobial mechanisms.

**TABLE 1:** Minimum inhibitory concentrations (MICs) of berberine against bacterial species.

Organism	Identifier	MIC (μg/mL)	References
*A. baumannii*	ATCC 19606	1,024	Li Y. et al., 2021
	Drug resistant (*n* = 4)	256–1,024	Li Y. et al., 2021
	CI (*n* = 5)	> 256	Ahmadi et al., 2022
*A. hydrophila*	**—**	125	Xue et al., 2015
*A. pleuropneumoniae*	**—**	312.5	Kang et al., 2015
*B. proteus*	ATCC 13315	256	Wen et al., 2016
*B. subtilis*	As.1.398	200	Jin et al., 2010
*C. acnes*	CI (*n* = 20)	5–25	Slobodníková et al., 2004
*C. difficile*	RT012	1,024	Wultańska et al., 2020
	ATCC 9689	256	Wultańska et al., 2020
	CI (*n* = 9)	256–900	Wultańska et al., 2020
*C. perfringens*	—	≈243.86	Yao et al., 2018
*C. violaceum*	ATCC 12472	2,500	Aswathanarayan and Vittal, 2018
	CV026	2,500	Aswathanarayan and Vittal, 2018
*E. coli*	DH10B	110/270	Li et al., 2018b,c
	KAM32	32	Xu et al., 2003
	ATCC 31343	2,000	Jin et al., 2010
	ATCC 25922	2,000	Jin et al., 2010
	STEC/EPEC (*N* = 5)	≈1.96 ± 0.11	Bandyopadhyay et al., 2013
	ETEC (*N* = 5)	≈1.80 ± 0.05	Bandyopadhyay et al., 2013
*E. faecalis*	ATCC 19 433	512	Gong et al., 2020
*E. typhosa*	ATCC 14028	512	Wen et al., 2016
*H. alvei*	CI (*n* = 1)	100	Pang et al., 2022
*H. pylori*	NCTC 11637	50	Li et al., 2018a
	Drug resistant (*n* = 4)	25,000–100,000	Huang et al., 2015
*K. pneumoniae*	CI (*n* = 9)	2,000	Magesh et al., 2013
	Drug resistant (*n* = 20)	512–> 512	Zhou et al., 2016
*L. monocytogenes*	CMCC 54004	8,129	Liu et al., 2015
*M. abscessus*	—	250	Tseng et al., 2020
*M. aurum*	ATCC 23366	62.5	Wijaya et al., 2022
*M. avium*	ATCC 25291	31.25	Wijaya et al., 2022
*M. luteus*	ATCC 4698	512	Wen et al., 2016
*M. kansasii*	ATCC 12478	31.25	Wijaya et al., 2022
*M. smegmatis*	ATCC 607	62.5	Wijaya et al., 2022
*M. tuberculosis*	ATCC 9431	125	Wijaya et al., 2022
*N. gonorrhoeae*	FA19	2.5	Rouquette-Loughlin et al., 2003
NTM	**—**	128–512	Puk and Guz, 2022
*N. meningitidis*	NMB	80	Rouquette-Loughlin et al., 2003
*P. aeruginosa*	PA01	1,250	Aswathanarayan and Vittal, 2018
	PA01-JP2	156	Aswathanarayan and Vittal, 2018
	As1.50	200	Jin et al., 2010
	ATCC 27853	≥ 128	Liu et al., 2024
	CICC 10351	128	Yuan et al., 2016
	CI (*n* = 60)	125–250	Aghayan et al., 2017
*P. vulgaris*	CICC 22929	256	Yuan et al., 2016
	As1.491	1,000	Jin et al., 2010
*Salmonella*	CI (n = 10)	> 625–1,250	Cui et al., 2024
	Drug resistant (*n* = 1)	3,125	Shi et al., 2018
*S. agalactiae*	CVCC 1886	0.78	Peng et al., 2015
*S. aureus*	ATCC 25923 (MSSA)	125	Mohtar et al., 2009
	NCTC 8325 (MSSA)	256	Zhang et al., 2022
	ATCC 35931 (MRSA)	> 250	Mohtar et al., 2009
	N315 (MRSA)	256	Zhang et al., 2022
	USA300 (MRSA)	256	Zhang et al., 2022
*S. aureus*	ATCC 33591 (MRSA)	128	Chu et al., 2016
	XN108/Mu50	> 512	Zhang et al., 2022
	CI (MRSA, *n* = 43)	32–128	Yu et al., 2005; Liang et al., 2014
	CI (*n* = 60)	12–512	Tocci et al., 2013; Tan et al., 2019
*S. boydii*	ATCC 8700	35	Joshi et al., 2011
*S. capitis*	ATCC 35661	16	Wojtyczka et al., 2014
*S. dysenteriae*	LMP 0208U	100	Joshi et al., 2011
*S. enteritidis*	**—**	500	Iwasa et al., 1998; Yao et al., 2018
*S. epidermidis*	ATCC 12228	32	Wojtyczka et al., 2014
	ATCC 35983	128	Wang et al., 2009b
	CI	256	Wang et al., 2009b
	CI (*n* = 14)	25–> 500	Slobodníková et al., 2004
*S. flexneri*	MTCC 1457	40	Joshi et al., 2011
	SF301	640	Fu et al., 2010
*S. galinarium*	ATCC 700401	128	Wojtyczka et al., 2014
*S. haemolyticus*	ATCC 29970	256	Wojtyczka et al., 2014
*S. hominis*	ATCC 27844	64	Wojtyczka et al., 2014
*S. intermedius*	ATCC 29663	64	Wojtyczka et al., 2014
*S. lentus*	ATCC 700403	64	Wojtyczka et al., 2014
*S. lugdunensis*	ATCC 49576	64	Wojtyczka et al., 2014
*S. mutans*	ATCC 25175	1,024	Dziedzic et al., 2015
*S. oralis*	ATCC 9811	1,024	Dziedzic et al., 2015
*S. pneumoniae*	DP1004	11	Tocci et al., 2013
	ATCC 49619	256	Yuan et al., 2016
*S. pyogenes*	MGAS 5005	80	Du et al., 2020
	CICC 10464	> 256	Yuan et al., 2016
*S. sanguinis*	ATCC 10556	512	Dziedzic et al., 2015
*S. saprophyticus*	ATTC 15303	512	Wojtyczka et al., 2014
*S. sciuri*	ATCC 29060	128	Wojtyczka et al., 2014
*S. simulans*	ATCC 27851	128	Wojtyczka et al., 2014
*S. sonnei*	MTCC 2957	50	Joshi et al., 2011
*S. typhi*	CI	6	Wu et al., 2005
*S. typhimurium*	CI	76	Liu et al., 2024
	SL1344	2,048	Liu et al., 2015
	CMCC 50115	900	Xu et al., 2021
	As1.1174	2,000	Jin et al., 2010
*S. warneri*	ATCC 49454	512	Wojtyczka et al., 2014
*S. xylosus*	ATCC 700404	128	Wojtyczka et al., 2014
*Y. pestis*	**—**	2,500	Zhang et al., 2009

*A. baumannii*, *Acinetobacter baumannii*; *A. hydrophila*, *Aeromonas hydrophila*; *B. proteus*, *Bacillus proteus*; *A. pleuropneumoniae*, *Actinobacillus pleuropneumoniae*; *B. subtilis*, *Bacillus subtilis*; *C. acnes, Cutibacterium acnes*; *C. difficile*, *Clostridioides difficile*; *C. perfringens*, *Clostridium perfringens*; *C. violaceum*, *Chromobacterium violaceum*; *E. coli*, *Escherichia coli*; *E. faecalis*, *Enterococcus faecalis*; *E. typhosa*, *Eberthella typhosa*; *H. alvei*, *Hafnia alvei*; *H. pylori*, *Helicobacter pylori*; *K. pneumoniae*, *Klebsiella pneumoniae*; *L. monocytogenes*, *Listeria monocytogenes*; *M. abscessus*, *Mycobacterium abscessus*; *M. aurum*, *Mycolicibacterium aurum*; *M. avium*, *Mycobacterium avium*; *M. luteus*, *Micrococcus luteus*; *M. kansasii*, *Mycobacterium kansasii*; *M. smegmatis*, *Mycolicibacterium smegmatis*; *M. tuberculosis*, *Mycobacterium tuberculosis*; *N. gonorrhoeae*, *Neisseria gonorrhoeae*; NTM, non-tuberculous mycobacteria; *N. meningitidis*, *Neisseria meningitidis*; *P. aeruginosa*, *Pseudomonas aeruginosa*; *P. vulgaris*, *Proteus vulgaris*; *S. agalactiae*; *Streptococcus agalactiae*; *S, aureus*, *Staphylococcus aureus*; *S. boydii*, *Shigella boydii*; *S. capitis*, *Staphylococcus capitis*; *S. dysenteriae*, *Shigella dysenteriae*; *S. enteritidis*, *Salmonella enteritidis*; *S. epidermidis*, *Staphylococcus epidermidis*; *S. flexneri*, *Shigella flexneri*; *S. galinarium*, *Staphylococcus galinarium*; *S. haemolyticus*, *Staphylococcus haemolyticus*; *S. hominis*, *Staphylococcus hominis*; *S. intermedius*, *Staphylococcus intermedius*; *S. lentus, Staphylococcus lentus*; *S. lugdunensis*, *Staphylococcus lugdunensis*; *S. mutans, Streptococcus mutans*; *S. oralis*, *Streptococcus oralis*; *S. pneumoniae*, *Streptococcus pneumoniae*; *S. pyogenes*, *Streptococcus pyogenes*; *S. sanguinis*, *Streptococcus sanguinis*; *S. saprophyticus*, *Staphylococcus saprophyticus*; *S. sciuri*, *Staphylococcus sciuri*; *S simulans*, *Staphylococcus simulans*; *S. sonnei*, *Shigella sonnei*; *S. typhi*, *Salmonella typhi*; *S. typhimurium*, *Salmonella typhimurium*; *S.warneri, Staphylococcus warneri*; *S. xylosus*, *Staphylococcus xylosus*; *Y. pestis*, *Yersinia pestis*; CI, clinical isolates.

Another notable characteristic of berberine is the low likelihood of pathogens developing resistance to it. Studies have shown that after 200 generations of exposure to berberine, the MIC of *E. coli* remained unchanged, while the MICs of neomycin and cefotaxime increased more than 10-fold (Jin et al., 2010). As an amphipathic cation, berberine is a natural substrate for bacterial efflux pumps, which are among the most critical mechanisms of resistance (Seukep et al., 2020). This property renders existing resistance mechanisms less likely to affect berberine’s activity. Consequently, berberine can act as an antibiotic adjuvant, competitively binding to efflux pumps and reducing drug efflux, thereby enhancing the antimicrobial activity of other antibiotics. However, it is important to note that berberine’s effects on efflux pumps vary across species. Recent studies found that low concentrations of berberine promoted the growth and resistance of *Klebsiella pneumoniae* by upregulating the expression of the efflux pump *KmrA*, while higher concentrations inhibited its growth (Li Y. et al., 2021). Similarly, overexpression of the efflux pump *HmrM* in *Haemophilus influenzae* resulted in an elevated MIC for berberine (Xu et al., 2003). In contrast, in *P. aeruginosa*, berberine reduced AMR by inhibiting the MexXY-OprM efflux pump (Su and Wang, 2018). This complex interaction is consistent with the varying antimicrobial activities of berberine against different strains. Additionally, low doses of berberine have been reported to have mitohormesis, offering protective benefits to neuroprotective cells (Zhu et al., 2020). Indeed, low concentration of berberine also promotes the growth of *C. difficile* biofilms, as well as *Enterobacter cloacae* and *A. baumannii* (Wultańska et al., 2020; Li Y. et al., 2021). Therefore, although berberine exhibits significant antimicrobial activity, the potential toxicity at low doses and the impact of drug efflux pumps on its efficacy should still be considered.

### 3.2 Toxicity-modulating effects of berberine

Virulence factors such as adhesion, biofilms, toxins, and quorum-sensing molecules not only help pathogenic bacteria evade host immune surveillance to promote colonization, but also synergistically invade host cells to cause damage (Lu et al., 2024). Berberine has been reported to directly inhibit the production of enterotoxin in certain *Vibrio cholerae* and *E. coli* for the treatment of bacterial diarrhea (Fu et al., 2010). In *Aeromonas hydrophila*, berberine similarly inhibits endotoxin and hemolysin secretion in a dose-dependent manner, reducing its hemolytic activity (Xue et al., 2015). Recent studies have also shown that berberine can inhibit the activities of pyocyanin and urease, thereby reducing the virulence and colonization of *P. aeruginosa* and *H. pylori* (Li et al., 2018a; Zhao et al., 2022). Our study also demonstrated that that subinhibitory concentrations of berberine reduce the production of *C. difficile* toxins TcdA and TcdB by inhibiting toxin synthesis genes, thereby decreasing its cellular invasiveness (Yang et al., 2025). In addition to directly inhibiting toxin production, berberine also exhibits significant anti-adhesion and anti-invasion properties. Berberine was found to inhibit adhesion and migration of HEp-2 cells induced by *Chlamydia pneumoniae* infection, thereby reducing the invasive power of HEp-2 cells (Zhang et al., 2011). In bacterial infections, berberine (20 μg/mL) reduced *Salmonella Typhimurium* adhesion and invasion of colon cells by 54.86% and 55.37%, respectively (Aswathanarayan and Vittal, 2018). Moreover, berberine could attenuate the adhesion and intracellular invasion of *MRSA* on epithelial cells and reduce its induced apoptosis in a dose-dependent manner (Yu et al., 2005; Xiong et al., 2014). Importantly, at concentrations effective against bacterial virulence, berberine does not exhibit toxicity to red blood cells, thymocytes, or splenocytes (Laudadio et al., 2019; Jhanji et al., 2021). Additionally, berberine downregulates the synthesis of staphyloxanthin by inhibiting the expression of the *S. aureus Fni* gene. Staphyloxanthin stabilizes the cell membrane by reducing membrane fluidity, enhancing its resistance to both host defenses and antibiotics (Qiu and Xu, 2024). *N*-acetyltransferase, associated with AMR in bacteria, promotes bacterial tolerance to aminoglycoside antibiotics. Berberine down-regulated *N*-acetyltransferase protein and gene expression in *S. aureus*, *H. pylori*, and *Salmonella typhi* in a dose-dependent manner (Wu et al., 2005; Wang et al., 2008; Chang et al., 2011).

Biofilms are critical virulence factors for many pathogenic microorganisms. Bacteria commonly adhere to host tissues such as carious teeth or the lungs of cystic fibrosis patients, or the surfaces of medical devices including artificial joints, heart valves, and urinary catheters, by forming biofilms. These structured microbial communities enhance antibiotic resistance, facilitate immune evasion, promote chronic infections, and can lead to secondary infections (Bouhrour et al., 2024). As shown in Table 2, berberine at concentrations ranging from 50 to 500 μg/mL significantly inhibited biofilm formation by clinically relevant pathogens, including *S. aureus*, *S. epidermidis*, *P. aeruginosa*, and *K. pneumoniae*, with inhibition rates exceeding 50% across all strains. In *S. aureus*, berberine not only inhibits biofilm formation in a dose-dependent manner but also interferes with the late-stage dispersal phase of biofilm development, thereby preventing the establishment of persistent bacterial colonies and reducing the risk of recurrent infections (Chu et al., 2016; Zhang et al., 2022). More importantly, studies have shown that a berberine-loaded liposomal hydrogel can effectively disrupt *S. aureus* biofilms and reduce biofilm biomass in infected mouse wounds, thereby promoting wound healing (Li S. et al., 2023). Another study demonstrated that when used as a root canal irrigant, berberine reduced bacterial counts by up to 99% in a multi-species dentin biofilm model containing *Fusobacterium nucleatum*, *E. faecalis*, and *Prevotella intermedia* (Xie et al., 2012). Wang et al. (2009a) also reported that berberin significantly inhibited the initial adhesion of *S. epidermidis* to titanium alloy disks (a common orthopedic implant material) within just 2 h, thereby preventing biofilm formation. Moreover, in biofilms formed by clinical isolates of *P. aeruginosa*, berberine significantly enhanced the antibacterial activity of tobramycin, reducing bacterial tolerance to the antibiotic by 10- to 1,000-fold (Mangiaterra et al., 2021). Shi et al. (2018) also found that berberine, when combined with ciprofloxacin, exerts a synergistic effect against biofilms formed by multidrug-resistant *Salmonella* strains by inhibiting the expression of the quorum-sensing system. Indeed, berberine disrupts biofilm formation and prevents dispersal of biofilm cells by downregulating related genes, inhibiting extracellular genomic DNA release and expression of polysaccharide intercellular adhesins, and interacting with quorum-sensing receptors (Guo et al., 2015; Zhou H. et al., 2023). Therefore, berberine shows significant potential in inhibiting bacterial biofilm formation and enhancing antibiotic sensitivity, offering a promising adjunctive strategy for the prevention and treatment of biofilm-associated infections.

**TABLE 2:** Inhibitory activity of berberine on biofilms.

Organism	Identifier	Concentration (μg/mL)	Inhibition rate (%)	References
*C. difficile*	ATCC 9689	128	0	Wultańska et al., 2020
	CI (*n* = 9)	128	0	Wultańska et al., 2020
*E. faecalis*	CI (*n* = 1)	100	> 80%	Chen et al., 2016
*H. alvei*	CI (*n* = 1)	50	40	Pang et al., 2022
*K. pneumoniae*	CI (*n* = 7)	62.5	75	Magesh et al., 2013
*M. abscessus*	–	250	> 70	Tseng et al., 2020
*P. aeruginosa*	PA01	128	> 85	Liu et al., 2024
*P. aeruginosa*	PA01	625	71.70	Aswathanarayan and Vittal, 2018
*Salmonella*	Drug resistant (*n* = 1)	1,562	61.4	Shi et al., 2018
*S. aureus*	ATCC 33593	64	36%	Seo et al., 2024
	ATCC 43300	1,024	71.80	Xia et al., 2022
	ATCC 33591	64	> 50	Chu et al., 2016
	ATCC 25923	500	> 50	Safai et al., 2022
	CI (MRSA)	512	95.65	Xia et al., 2022
	CI (*n* = 18)	128	> 60	Tan et al., 2019
	CI (*n* = 10)	> 1,024–1,024	100	Guo et al., 2015
*S. epidermidis*	ATCC 35984	60	100	Wang et al., 2009a
	ATCC 12228	60	100	Wang et al., 2009a
	SE 243	60	100	Wang et al., 2009a
*S. mutans*	ATCC 700610	64	> 70	Zhou H. et al., 2023
*S. typhimurium*	CI (*n* = 1)	19	31.20	Aswathanarayan and Vittal, 2018
*S. typhimurium*	CMCC 50115	56.25	66.29	Xu et al., 2021

### 3.3 Therapeutic efficacy of berberine in treating bacterial infections *in vivo*

Although the antibacterial activity of berberine has been well-characterized *in vitro*, limited *in vivo* studies have not fully elucidated its therapeutic potential. Table 3 provides a detailed summary of *in vivo* studies on berberine. Briefly, berberine exhibited a strong therapeutic effect on inflammatory responses induced by bacterial infections *in vivo*, whereas its direct antibacterial activity is comparatively weaker. For example, in *P. aeruginosa* infections, berberine reduces the bacterial burden in infected mice, but more noteworthy is its potent anti-inflammatory activity (Liu et al., 2024). Previous studies have also reported that berberine significantly reduces osteoclast recruitment and bone resorption, demonstrating a therapeutic effect on lipopolysaccharide-induced osteolysis (Zhou et al., 2012). Indeed, berberine can attenuate inflammatory responses, coagulation activation, and organ dysfunction caused by bacterial infections through multiple mechanisms, including inhibition of the caspase-11 pathway and inhibition of *COX-2* overexpression (Feng et al., 2012; Yuan et al., 2021). These aspects have been comprehensively reviewed by Izadparast et al. (2022), and the reader is referred to their work for more detailed information ( doi: 10.1080/15384101.2022.2100682). However, the antibacterial activity of berberine *in vivo* is relatively weaker compared to its *in vitro* efficacy. The probable reason for this is the low oral utilization and intestinal absorption of berberine and its very rapid blood clearance (Singh et al., 2021). After oral administration of 40 mg/kg berberine to mice, only trace amounts of berberine were detected in the plasma (Zuo et al., 2006). In human subjects, plasma berberine concentrations ranged from 1.23 to 2.10 ng/mL at 24 h after a 500 mg oral dose (Solnier et al., 2023). Although no side effects or adverse events were reported, such low plasma levels may prevent berberine from achieving effective antibacterial concentrations at infection sites, thereby limiting its clinical application. It is noteworthy that the cytotoxic threshold of berberine varies significantly across different cell lines: in L929 mouse fibroblast cells, cell viability decreases at concentrations as low as 50 μg/mL, whereas the half-maximal inhibitory concentrations in human HepG2 liver cells, NIH/3T3 fibroblasts, and 293T kidney cells are above 90, 100, and 80 μg/mL, respectively (Gu et al., 2015; Tong et al., 2021). Although *in vitro* results may not fully reflect *in vivo* conditions, its safety profile *in vivo* remains insufficiently characterized, particularly due to a lack of systematic evaluation of the effects of long-term or high-dose administration on major organs and tissues. Therefore, further investigation into the toxicological mechanisms of berberine *in vivo* is necessary to comprehensively clarify its safety. Nevertheless, as shown in Table 3, berberine exhibits significant synergistic effects with antibiotics *in vivo*, enhancing their antibacterial efficacy. In two randomized, open-label, non-inferiority clinical trials, a berberine-containing quadruple therapy demonstrated similar eradication rates and symptom improvement compared to conventional quadruple therapies for *H. pylori* infection (Zhang et al., 2017, 2020). In addition, berberine can alleviate drug-induced diarrhea and intestinal mucosal damage by modulating the intestinal microbiota, such as the anticancer agents irinotecan and 5-fluorouracil (Chen et al., 2020; Yue et al., 2021). Therefore, berberine holds promise as an antibiotic adjuvant for clinical antimicrobial therapy, helping to address the growing threat of AMR. Notably. antibiotics can negatively impact the gut microbiota, thereby reducing berberine’s bioavailability (Feng et al., 2015). Further studies on synergistic administration regimens of berberine and antibiotics are still needed in the future. Given berberine’s high tolerance and high LD50 (oral: 329 mg/kg, injection: 23 mg/kg) (Gasmi et al., 2024), along with its low propensity to induce resistance, increasing the berberine dose in combination therapy while reducing the antibiotic dose could be considered as a strategy to mitigate the occurrence of AMR.

**TABLE 3:** Antibacterial activity of berberine *in vivo*.

Species	Model	Animal	Dose	Route	Outcome	References
*A. baumannii*	Thigh infection	Mice (*N* = 5)	BBR+SUL (20 mg+/kg/12 h)	i.m.	SUL and BBH alone do not exhibit therapeutic effects; however, their combination demonstrates bactericidal activity against multidrug-resistant *A. baumannii* (*P* < 0.05).	Li Y. et al., 2021
*C. difficile*	–	Mice (*N* = 10)	BBR+VAN (100+50 mg/kg/d)	OG	Berberine alone can improve the survival rate of mice and reduce inflammatory infiltration. When combined with vancomycin, the effect is enhanced, and it also prevents the recurrence of *C. difficile* infection (*P* < 0.05).	Lv et al., 2015
CLP	Sepsis	Rat (*N* = 10)	BBR (50 mg/kg/d)	OG	Pre-treatment with berberine before septic infection improves the survival rate of rats, reduces plasma endotoxin levels, and alleviates hypozincemia in rats.	He et al., 2019
*E. coli/P. aeruginosa /S. aureus*	–	Zebrafish	BBR+RUT+SABX	i.m.	The combination therapy containing berberine significantly reduced bacterial load more than SABX alone (*P* < 0.05).	Jhanji et al., 2021
*E. coli*	Sepsis	Mice	BBR+IMI (5 + 20 mg/kg/8 h)	i.p.	Mice treated with berberine alone exhibited a survival rate of 50% at 24 h, which decreased to 20% at 48 h. However, pre-treatment with berberine followed by combination therapy with IMI resulted in complete survival of the mice (*P* < 0.05).	Pierpaoli et al., 2021
*E. coli*	–	*G. mellonella* (*N* = 20)	BBR (4,096 μg/mL)	–	Pre-treating *E. coli* with berberine or administering berberine to *G. mellonella* infected with *E. coli* significantly improved the survival rate of *G. mellonella* and reduced bacterial load (*P* < 0.05).	Petronio Petronio et al., 2020
*H. pylori*	Acute gastritis	Mice (*N* = 6)	BXXXD + OME (7/28+132.8 mg/kg/d)	OG	BXXXD combined with OME outperformed traditional triple therapy in reducing *H. pylori* colonization, suppressing inflammatory responses, and alleviating gastric mucosal damage (*P* < 0.001).	Ciccaglione et al., 2023; Li X. H. et al., 2023
*H. pylori*	Gastritis	Mice (*N* = 6)	CECY (100/200/400 mg/kg/d)	OG	High-dose CECY significantly inhibits the survival of *H. pylori* in the gastric mucosa, alleviates mucosal congestion and damage, reduces epithelial cell loss, and decreases IgG expression levels (*P* < 0.01).	Wu et al., 2023
*H. pylori*	Atrophic gastritis	Rat (*N* = 6)	BBR (14/28 mg/kg/d)	OG	Berberine could attenuate the histological damage of the gastric mucosa induced by. *H. pylori* exerted anti-inflammatory properties by inhibiting the IRF8-IFN-γ signaling axis (*P* < 0.01).	Yang et al., 2020
LPS	Endotoxemia	Mice /Rabbit (*N* = 10)	BBR (0.2 g/kg)/BBR (0.06 g/kg)	OG	Berberine treatment enhanced the survival rate following LPS infection and alleviated LPS-induced fever symptoms (*P* < 0.05).	Chu et al., 2014
*M. tuberculosis*	Tuberculosis	Mice	BBR+ISO+RIF (5.5 + 0.6 + 0.6 mg)	OG	Berberine alone or in combination with ISO and RIF does not affect pulmonary bacterial load; however, it can act as an immunomodulator to alleviate lung pathological changes (*P* < 0.05).	Ozturk et al., 2021
*P. aeruginosa*	Peritonitis model	Mice (*N* = 5)	BBR (20 μg/mL)	i.p.	Berberine treatment significantly reduced the intense inflammatory response (IL-6, IL-1β) and liver bacterial load induced by *P. aeruginosa*.	Liu et al., 2024
*P. aeruginosa*	lung infection	Mice (*N* = 8)	BBR+AZM (3.2+0.8 mg/kg)	T.v.i.	Berberine alone reduced bacterial load and inflammation in the lung tissues of infected mice, but the survival rate was only 1/8. In contrast, combination therapy with berberine and AMZ increased the survival rate to 7/8 and significantly reduced abscesses and hemorrhagic areas (*P* < 0.05).	Li et al., 2017
*Salmonella*	Intraperitoneal	Mice (*N* = 8)	BBR+COL+EDTA (80+8+10 mg/kg)	i.p.	The use of berberine alone slightly reduced the bacterial load in the liver and spleen of infected mice, whereas the triple therapy significantly decreased the bacterial load and restored the *in vivo* susceptibility to COL (*P* < 0.05).	Cui et al., 2024
*S. aureus*	Arthritis	Mice (*N* = 10)	BBR (50/100/200 mg/kg)	OG	Berberine significantly alleviates joint swelling and inflammatory responses caused by *S. aureus* (*P* < 0.05).	Asila et al., 2022
*S. typhimurium*	–	*C. elegans*	BBR (38 μg/mL)	–	Berberine dose-dependently reduced the paralysis rate in *C. elegans*, with a 65.38% reduction in paralysis (*P* < 0.05).	Aswathanarayan and Vittal, 2018
*S. typhimurium*	–	Mice (*N* = 10)	BBR (40 mg/kg)	OG	The survival rate of infected mice reached 90% after berberine treatment, compared to 50% in the untreated group (*P* < 0.05).	Chu et al., 2014
*S. typhimurium*	–	Mice (*N* = 10)	CR (250 mg/kg)	OG	CR can prevent weight loss and inflammatory responses caused by *S. typhimurium* infection, as well as reduce bacterial load (*P* < 0.05).	Chang et al., 2014

i.p., intraperitoneal injection; i.m., intramuscular injection; TA; topically applied, OG; oral gavage; T.v.i., tail vein injection; CLP, cecal ligation and puncture; BBR, berberine; COL, colistin; RUT, rutin; SABX, standard antibiotics; IMI, imipenem; AZM, azithromycin; OME, Omeprazole; BXXXD, BanXiaXieXin decoction (*Pinellia ternate*, *Radix scutellariae*, *Dried ginger*, *Ginseng*, *Roasted licorice*, *Coptis chinensis*, *Jujubes*); CECY, chloroform extracts of *Corydalis yanhusuo*; LPS, lipopolysaccharide; CR, *Coptidis rhizome*; SUL, sulbactam; ISO, isoniazid; RIF, rifampicin; VAN, vancomycin; *C. elegans*, *Caenorhabditis elegans*; *G. mellonella*, *Galleria mellonella*.

Overall, berberine exhibits dose- and time-dependent antimicrobial effects against clinically relevant pathogens, with its anti-inflammatory properties, low potential for resistance, and ability to mitigate drug side effects highlighting its potential as both an antimicrobial agent and antibiotic adjuvant. However, its low bioavailability, potential cytotoxicity, and lack of comprehensive *in vivo* evaluation hinder its clinical application. Future research should focus on addressing these challenges, particularly through systematic *in vivo* studies. Additionally, standardized evaluation methods are needed to resolve the MIC discrepancies observed in current studies, with the approach proposed by Alharthi et al. (2021) providing a solution ( doi: 10.1016/j.bmc.2021.116527).

## 4 Antibacterial mechanisms of berberine

### 4.1 Berberine inhibits bacterial division by targeting the FtsZ protein

Filamentous temperature-sensitive mutant Z (FtsZ) is a key organizer of bacterial cell division. During the division process, FtsZ associates with membrane-associated proteins and assembles into protofilaments through GTP-dependent polymerization, forming a Z-ring that ensures the correct localization of other division proteins such as FtsA and ZipA (Cameron and Margolin, 2024). Berberine can target FtsZ to inhibit bacterial growth. In *E. coli*, berberine significantly reduces Z-ring formation, and silencing the FtsZ gene enhances bacterial sensitivity to berberine, reducing its MIC by 2-fold. Conversely, overexpression of FtsZ increases resistance to berberine (Boberek et al., 2010). Consistently, another study demonstrated that berberine treatment severely disrupts *E. coli* cell division, resulting in significantly elongated cells (Budeyri Gokgoz et al., 2017). Berberine spontaneously binds to the GTP-binding pocket of FtsZ in a dose-dependent manner and a 1:1 ratio, inhibiting FtsZ monomer interactions and disrupting the formation of FtsZ protofilaments. This results in the mislocalization and spatial disorganization of the Z-ring, thereby hindering cell division (Domadia et al., 2008). Notably, FtsZ is highly conserved and widely present across various bacterial species. In *B. anthracis*, *MRSA*, and *E. faecium*, berberine also exhibits significant inhibitory effects on the GTPase and polymerization activities of FtsZ (Park et al., 2014; Sun et al., 2014). Through virtual screening and computational methods, recent studies have revealed that berberine can form stable complexes with the FtsZ of *Mycobacterium tuberculosis* and *Salmonella typhi*, demonstrating high binding affinity (Akinpelu et al., 2022; Naz et al., 2022). This may explain the broad-spectrum antibacterial activity and dose-dependent inhibitory effects exhibited by berberine.

### 4.2 Berberine targets bacterial cell membranes and walls to disrupt cell structure

The cell membranes and cell walls are primary targets for existing antibiotics. For instance, β-lactam antibiotics prevent the cross-linking of peptidoglycan in the bacterial cell wall. Peptide antibiotics interfere with cell membrane synthesis by inhibiting lipid integration into the cell membrane (Baran et al., 2023). Berberine, however, binds to cell membranes and cell walls by a mechanism of action different from the above, thereby inhibiting bacterial growth. Due to the lack of extensive hydrogen bonding, berberine, in its positively charged form, can intercalate into lipid bilayers and penetrate the cell interior. Nevertheless, it also disrupts the phospholipid bilayer (Sokolov et al., 2023). Upon exposure to berberine, bacteria such as *P. aeruginosa*, *S. agalactiae*, and *A. pleuropneumoniae* exhibit features of membrane lysis and cell wall damage, including cytoplasmic shrinkage and leakage of cell contents (Kang et al., 2015; Peng et al., 2015; Liu et al., 2024). Recent studies have shown that berberine increases membrane permeability in *MRSA* in a dose-dependent manner and directly adheres to the bacterial cell wall, disrupting its structure and leading to cell lysis (Zhou F. F. et al., 2023). Similar alterations in cell surface structure were observed in *E. coli*, accompanied by the release of Ca^2 +^ and K^+^ ions (Jin et al., 2010). Indeed, berberine can directly bind to cell wall components, such as *lipopolysaccharides* and *peptidoglycans*, disrupting normal cell wall physiological processes (Li et al., 2018c). Notably, in fungi, berberine also damages the cell membrane by inhibiting enzymes and downregulating genes involved in ergosterol synthesis (Ding et al., 2024). However, bacteria like *E. coli* upregulate genes related to cell wall and membrane transport and synthesis after berberine exposure (Zhang et al., 2009; Karaosmanoglu et al., 2014). This response may represent a stress reaction to membrane and wall damage, but also indicates that berberine does not inhibit the expression of these genes to disrupt the cell membrane. As previously mentioned, berberine is a natural substrate of efflux pumps, which increase membrane potential by exporting protons, thereby attracting positively charged molecules such as berberine. Zhao et al. (2023) demonstrated that berberine efflux via drug efflux pumps dissipates membrane potential, resulting in increased intracellular accumulation of berberine and heightened membrane instability. This may also explain the time-dependent antibacterial activity of berberine and its effects on the cell membrane. However, due to the effect of the efflux pump, this can also lead to a decrease in bacterial sensitivity to berberine, as has been demonstrated in several studies (Xu et al., 2003; Li et al., 2018b). In summary, berberine primarily exerts its effects on bacterial cell walls and membranes through its physical properties, disrupting the normal structure of the cell.

### 4.3 Berberine inhibits the fundamental metabolic processes of bacteria

Berberine has a high affinity for DNA and RNA. It causes DNA damage by inserting into the DNA structure and forming strong interactions through hydrogen bonding, van der Waals forces, and electrostatic forces (Budeyri Gokgoz et al., 2017). Studies have shown that berberine exerts anticancer activity by disrupting cell division and inducing apoptosis through binding to histone-DNA complexes (Sokolov et al., 2023). In bacteria, berberine not only binds to DNA and RNA, causing damage, but also inhibits essential biological processes such as DNA, RNA, and protein synthesis. Binding kinetics indicate that berberine readily binds to and remains tightly bound to DNA and RNA in *E. coli*, thereby inhibiting DNA replication, RNA transcription, and protein synthesis to exert antibacterial activity (Jin et al., 2010). Consistently, in *A. pleuropneumoniae* and *S. agalactiae*, berberine reduced DNA and protein levels in a time-dependent manner, probably due to its gradual accumulation within the cell (Kang et al., 2015; Peng et al., 2015). In addition, berberine disrupts the redox homeostasis of bacteria, generating reactive oxygen species (ROS) that attack key cellular components such as DNA, membranes, and mitochondria. Avci et al. (2019) reported that berberine induced oxidative stress and intracellular accumulation of reactive substances in *E. coli*. Similarly, elevated ROS levels were observed in *P. aeruginosa* and *Streptococcus pyogenes* after treatment with berberine, and co-culture with antioxidants partially attenuated their antimicrobial activity (Du et al., 2020; Liu et al., 2024). In fungi, mitochondrial dysfunction caused by ROS is the main antifungal mechanism of berberine (Ding et al., 2024). Although berberine has been reported to inhibit intracellular ATP production (Liu et al., 2024), its specific effect on bacterial mitochondrial function remains unclear and deserves further investigation. Nevertheless, ROS production still contributes to the antibacterial activity of berberine. However, bacterial DNA damage triggers the SOS response (a post-replicative DNA repair system), which inhibits bacterial division. In *E. coli*, berberine inhibits bacterial division in wild-type and SOS-negative strains, while SOS-negative strains do not respond to SOS-induced inhibition of cell division (Boberek et al., 2010). Furthermore, recent studies have found that only about 5% of the berberine accumulated in *S. aureus* cells binds to DNA (Zhao et al., 2023). This suggests that the primary mechanism of berberine’s inhibition of bacterial division involves targeting FtsZ, while its effects on DNA, RNA, and proteins predominantly influence bacterial metabolic processes. In various bacteria, including *E. coli*, *Yersinia pestis*, and *S. flexneri*, significant changes have been observed in metabolic pathways such as carbohydrate metabolism, energy production and conversion, DNA replication and repair, pyrimidine metabolism, RNA degradation, and ribosome function (Zhang et al., 2009; Fu et al., 2010; Budeyri Gokgoz et al., 2017). Notably, DNA replication, repair, and pyrimidine metabolism are significantly upregulated in response to berberine-induced DNA damage. Thus, berberine exerts its antibacterial activity synergistically by targeting key biomolecules and disrupting essential bacterial metabolic processes.

In conclusion, berberine exhibits antibacterial activity through a multifaceted mechanism, including targeting FtsZ, disrupting the cell membrane and cell wall, and interacting with DNA, RNA, proteins, and bacterial redox homeostasis. This multi-target mode of action not only disrupts fundamental bacterial processes but also hinders the development of resistance to berberine. However, the adverse effects of drug efflux pumps significantly limit the application of berberine by reducing its intracellular accumulation and thus diminishing its antibacterial efficacy. Therefore, future research aimed at overcoming efflux pump-mediated resistance holds promise for enhancing the therapeutic potential of berberine.

## 5 Future avenues of application for berberine

### 5.1 Synergistic antibacterial activity and mechanisms of berberine-containing natural extracts

Berberine is found in various medicinal plants, including *Hydrastis canadensis*, *Berberis aristata*, *Coptis chinensis*, and *Coptis rhizome* (Zhou H. et al., 2023). These plants are distributed globally and offer significant advantages such as accessibility and low cost. More importantly, the extracts from these plants also exhibit direct antimicrobial activity. Supplementary Table 1 (Antibacterial activity of berberine (BBR) extracts against bacteria) summarizes the antimicrobial activity of berberine extracts. In brief, berberine extracts display antimicrobial activity similar to or even superior to that of pure berberine, although the results are not universally consistent. For instance, in the same study, *Hydrastis canadensis* extract had an MIC of 15 mg/mL against *P. aeruginosa*, whereas the MIC of berberine was > 120 mg/mL, and the reverse was observed for *S. aureus* (Scazzocchio et al., 2001). This differential effect may be related to the mode of interaction between berberine and other active ingredients in the extract. Previous studies have demonstrated that 5′-methoxyhydnocarpin isolated from *Berberis fremontii* can inhibit drug efflux pumps, thereby increasing the intracellular accumulation of berberine and reducing its MIC against *S. aureus* by 8-fold (Stermitz et al., 2000). Moreover, the extracts of *Lupinus argenteus* and *Hydrastis canadensis L.* also demonstrate synergistic effects with berberine (Morel et al., 2003; Ettefagh et al., 2011). Table 4 summarizes other plant compounds that exhibit synergistic effects when combined with berberine. While no antagonistic effects have been reported with berberine, it is plausible that such compounds may exist in plant extracts. Notably, antimicrobial activity varies among different parts of the same plant. For example, the MIC of *Berberis microphylla* root extract against *S. aureus* is 2–3 times higher than that of its leaf and stem extracts (Manosalva et al., 2016). Furthermore, plant extracts also exhibit *in vivo* anti-inflammatory and antimicrobial activities, as outlined in Table 3. Recent studies have demonstrated that *Coptis chinensis* extract inhibits the production of pro-inflammatory cytokines such as TNF-α, IL-1β, and the NF-κB signaling pathway induced by *Propionibacterium acnes*, showing its potential for treating acne-related inflammatory skin conditions (Lee et al., 2018). In another study, *Berberis aristata* extract demonstrated not only *in vitro* antibacterial activity against *Shigella* but also exhibited antidiarrheal activity *in vivo*, with an LD_50_ > 5,000 mg/kg (Joshi et al., 2011). Given that these plants have been used in traditional ethnomedicine for centuries, their extracts possess great therapeutic potential in combating antimicrobial infections, potentially offering a promising strategy to address the escalating global threat of AMR.

**TABLE 4:** The effect of the combination of berberine (BBR) and natural components on the minimum inhibitory concentrations (MICs) of bacterial.

Species	Identifier	Alone (μg/mL)		In combination (μg/mL)		FICI	Type	References
		NC	MIC	Berberine	NC			
*B. subtilis*	DSM 402	Carvacrol	200	75	25	−	S	Atas et al., 2022
*E.coli*	OQ600604.1	Matrine	6,250	62.5	1562.5	0.3125	S	Meng et al., 2024
*MRSA*	CI (n = 124)	—	+	32–512	8–256	−	S	Li et al., 2020
S.aureus	ATCC 25923	Thymol	256	32	64	0.5	S	Aksoy et al., 2020
				64	32	0.625	A	
				8	128	1.03	I	
*S.aureus in suspension*	CI (*n* = 11)	Totarol	0.5–2/2–16	8–32	0.0625–1	0.313–1	S or I	Guo et al., 2015
*S.aureus in biofilm*	CI (*n* = 11)	Totarol	2–8/32–1,024	8–64	0.125–1	0.188–0.500	S	
*s.aureus*	—	Flavone1	250–500	25	30	−	S	Stermitz et al., 2002
	—	Flavone2	250–500	6.25	30	−	S	
*S. aureus*	8325-4	Isoflavone	+	+	10	−	S	Morel et al., 2003
*B. megaterium*	1,1561	Isoflavone	+	+	10	−	S	

*B. megaterium*, *Bacillus megaterium*; NC, natural component.

### 5.2 Antibacterial activity and physical properties of berberine nanoparticles

Although berberine has potential cytotoxicity and poor bioavailability, its combination with nanotechnology can overcome these limitations. As summarized in Supplementary Table 2 (MICs of berberine nanoformulations against bacteria), when berberine is combined with nanocarrier systems such as liposomes, shellac, and metal ions, it exhibits improved biocompatibility, low toxicity, high bioavailability, and enhanced antimicrobial activity. Compared to free berberine, lipid-reconstituted nanoparticle-coated poly (lactic-co-glycolic acid) nanoparticles loaded with berberine have a significantly lower MIC of 5 μg/mL against *Mycobacterium smegmatis*, whereas the MIC of free berberine is 100 μg/mL (Pu et al., 2024). Al-Obaidy et al. (2019) also observed that dual-functionalized shellac nanocarriers can enhance the local concentration of berberine, thereby improving its biological stability and bioavailability. In another study, gold nanoparticles were shown to double the antimicrobial activity of berberine against *S. aureus* biofilms. In an infected skin model, berberine-loaded gold nanoparticles reduced the survival rate of MRSA to only 2.7%, with no observed toxicity in mouse fibroblast cells (Sadeghi et al., 2024). In addition, the physical and chemical properties of different nanocarriers significantly influence their performance in drug delivery systems and *in vivo* applications. For example, liposomes encapsulating berberine achieve an encapsulation efficiency of up to 69.8% (Pu et al., 2024). In contrast, shellac and metal-organic frameworks (MOFs) exhibit lower encapsulation efficiencies of approximately 60% and 35%, respectively. However, under near-physiological pH conditions, shellac and MOFs demonstrate higher drug release rates, reaching up to 80%, whereas liposomes release only 57.3% of the encapsulated drug (Al-Obaidy et al., 2019; Hu et al., 2023; Pu et al., 2024). Furthermore, liposomal encapsulation of berberine can improve its bioavailability by prolonging its *in vivo* retention time (Sun et al., 2024). According to the findings of Abo El-Enin et al. (2022), the concentration of berberine at the target site was 13.2 times higher in the liposome-treated group compared to the control group, indicating significantly enhanced targeting efficiency. The shellac-based delivery system exhibits strong adhesion to microbial cell walls, which further improves targeting and enhances antimicrobial activity (Sun et al., 2024). Remarkably, MOFs have demonstrated pronounced advantages in targeted delivery. For instance, Wang et al. (2017) developed magnetic mesoporous silica nanoparticles capable of controlled drug release under an external magnetic field. However, the elemental composition and surface charge of metallic nanoparticles may increase their toxicity (Sun et al., 2024). Despite these promising findings, many types of nanocarriers still lack comprehensive *in vivo* evaluations. Therefore, further preclinical and clinical investigations are warranted to substantiate their safety and therapeutic efficacy. Moreover, drug self-assembled nanoparticles, which do not require carriers, not only retain these advantages but also exhibit higher drug-loading capacity. For example, self-assembled nanoparticles of berberine and flavonoids show enhanced affinity for *S. aureus*, leading to bacterial collapse and reduced biofilm formation, while demonstrating good biocompatibility in zebrafish toxicity assessments (Li et al., 2019). Recent studies have also shown that gallic acid and berberine nanoparticles, formed through electrostatic interactions, π-π stacking, and hydrophobic interactions, exhibit antimicrobial and anti-biofilm activities in a *S. aureus* wound infection model, along with potent anti-inflammatory and pro-angiogenic effects (Chen et al., 2023). Therefore, utilizing the unique properties of nanomaterials can enhance the antimicrobial efficacy of berberine *in vivo*, offering an alternative strategy to combat the growing threat of AMR. However, the research on self-assembly still has problems such as preparation stability, which will be a direction for subsequent research.

In summary, berberine extracts and nanomaterial-based formulations offer distinct advantages in antibacterial therapy. Berberine extracts have a long history of use in treating inflammation and bacterial diarrhea, with promising applications in combating bacterial infections. In contrast, berberine nanomaterials exhibit enhanced bioavailability and lower toxicity, further improving both efficacy and biological safety. However, comprehensive *in vivo* studies are still lacking. Moreover, the significant antibacterial activity exhibited by berberine derivatives provides new avenues for its further development, as systematically summarized by Xiao et al. (2018), Jamshaid et al. (2020).

## 6 Conclusion and perspective

To address the growing global threat of AMR, natural bioactive compounds offer a promising therapeutic strategy. Compared to existing single-target antimicrobial drugs, the natural active compound berberine not only exhibits a multi-target mechanism of action against bacteria, but also has lower toxicity, fewer side effects, and offers beneficial effects by reducing the adverse reactions associated with antibiotics. Importantly, not only are berberine-containing medicinal plants widely distributed and traditionally used throughout the world, but the extraction of berberine from medicinal plants is also consistent with healthcare economics. These factors highlight the significant potential of berberine as an antimicrobial agent. However, the clinical application of berberine is significantly limited by factors such as potential cytotoxicity, low bioavailability, insufficient systematic *in vivo* evaluation of its antimicrobial activity, and the impact of drug efflux pumps. Although combining with nanotechnology may improve the above-mentioned drawbacks of berberine, systematic *in vivo* validation is lacking. More importantly, there is a lack of systematic research and in-depth discussion on the current status of clinical trials and the regulatory landscape of berberine in the field of antibacterial therapy, which significantly limits its clinical translation for infectious diseases. In the current context, the clinical use of berberine may be limited to its use as an antibiotic adjuvant against AMR bacterial infections or the use of berberine decoction for the treatment of mild infections such as skin and mucous membrane infections. Future research should focus on optimizing berberine-based formulations and conducting systematic *in vivo* and clinical studies to thoroughly evaluate its long-term safety, *in vivo* efficacy, and clinical applicability, thereby advancing the clinical application of berberine. To date, no plant-derived active compound has successfully passed clinical trials. Further research on berberine may pave the way for the application of plant-derived compounds in antimicrobial therapy. Achieving this milestone requires the collective effort of researchers, but it remains the ultimate goal for pharmacologists and microbiologists.
