# Supplementary material for: Berberine and its nanoformulations and extracts: potential strategies and future perspectives against multi-drug resistant bacterial infections
Source: Front Microbiol. 2025 Sep 2;16:1643409. doi: 10.3389/fmicb.2025.1643409 (PMC12436466; doi:10.3389/fmicb.2025.1643409)
Supplement: Supplementary file 1 [file Data_Sheet_1.zip › Supplementary Table 2.docx]

**Table S2 MICs of berberine nanoformulations against bacteria**

| **Nano-formulations** | **Species** | **Identifier** | **MIC (μg/mL)** | | **Properties** | **References** |
| --- | --- | --- | --- | --- | --- | --- |
|  |  |  | **Free Berberine** | **Encapsulated Berberine** |  |  |
| AgNPs | *A. baumannii* | ATCC 19609 | + | 4 | Antibacterial; Non-toxic | (Tahan et al., 2024) |
|  | *P. aeruginosa* | ATCC 27853 | + | 1 | Antibacterial; Non-toxic |  |
| AuNP-conjugated BER | *S. aureus* | ATCC 6538 | 82.5 | 27.37 | Antibacterial; Antibiofilm  Non-toxic | (Sadeghi et al., 2024) |
|  | *MRSA* | CI (n=4) | 165 | 109.5 | Antibacterial; Antibiofilm  Non-toxic |  |
| BBC–ZnAl-LDH [biohybrids](https://www.sciencedirect.com/topics/chemistry/biohybrids) | [*S. aureus*](https://www.sciencedirect.com/topics/agricultural-and-biological-sciences/staphylococcus-aureus) | CIP 543154 | + | + | Antibacterial | (Djebbi et al., 2016) |
|  | [*P. aeruginosa*](https://www.sciencedirect.com/topics/pharmacology-toxicology-and-pharmaceutical-science/pseudomonas-aeruginosa) | A22 | + | + | Antibacteria |  |
|  | *B. subtilus* | ILP 1428B | + | + | Antibacterial |  |
| BBR-loaded SeNPs | *K. pneumoniae* | CI (n=100) | + | + | Antibacterial | (Hasan et al., 2023) |
| BBR/CGA nanoparticles | *MRSA* | — | 2 μmol/mL | 1.5 μmol/mL | Antibacterial; Antibiofilm |  |
|  | *S. aureus* | — | 2 μmol/mL | 1.5 μmol/mL | Antibacterial; Antibiofilm |  |
| BBR-EGCG NPs | *S. aureus* | — | 62.50 | 50 | Antibacterial; Antibiofilm | (Zheng et al., 2023b) |
|  | *MRSA* | — | >62.50 | 50 | Antibacterial; Antibiofilm |  |

**Table S2 Continued**

| **Nano-formulations** | **Species** | **Identifier** | **MIC (μg/mL)** | | **Properties** | **References** |
| --- | --- | --- | --- | --- | --- | --- |
|  |  |  | **Free Berberine** | **Encapsulated Berberine** |  |  |
| BBR-PLGA-LrNs | *M. smegmatis* | — | 100 | 5 | Antibacterial | (Zheng et al., 2023b) |
| BBR-TA NPs | *S. aureus* | — | 62.50 | 15.63 | Antibacterial | (Zheng et al., 2024) |
|  | *MRSA* | 19PXTH0119 | 62.50 | 15.63 | Antibacterial |  |
| Berberine-loaded fucose-conjugated NP system | *H. pylori* | ATCC 700392 | + | 6000 | Antibacterial | (Lin et al., 2015) |
| BH-CMC hydrogel | *S. aureus* | ATCC 29740 | + | + | Antibacterial | (Tong et al., 2022) |
|  |  | ATCC 29213 | + | + | Antibacterial |  |
| BH@MTN-PEI | *MRSA* | — | — | — | Antibiofilm | (Ning et al., 2022) |
| BR-loaded NPs | *MRSA* | ATCC 43300 | + | 125 | Antibacterial | (Alharthi et al., 2023) |
|  | *S. aureus* | ATCC 25923 | + | 125 | Antibacterial |  |
|  | *E. coli* | ATCC 25922 | + | 250 | Antibacterial |  |
|  | *P. aeruginosa* | ATCC 27853 | + | 500 | Antibacterial |  |
| BRB-loaded shellac NPs | *C. reinhardtii* | — | + | + | Antibacterial |  |
| CABs | Bacteria | — | + | + | Antibacterial | (Zheng et al., 2023a) |

**Table S2 Continued**

| **Nano-formulations** | **Species** | **Identifier** | **MIC (μg/mL)** | | **Properties** | **References** |
| --- | --- | --- | --- | --- | --- | --- |
|  |  |  | **Free Berberine** | **Encapsulated Berberine** |  |  |
| CA-BBR NPs | *MRSA* | — | 0.1 μmol/mL | 0.075 μmol/mL | Antibacterial | (Huang et al., 2020) |
| Co-amorphous PTM-BCL | *MRSA* | — | 64 | + | Antibacterial | (Wang et al., 2022) |
| Combinatorial liposomes of  *berberine* and *curcumin* | *S. aureus* | ATCC 43300 | + | + | Antibacterial; Antibiofilm | (Bhatia et al., 2021) |
| Cu @ Bbc | *MRSA* | — | + | + | Antioxidant; Antibacterial  Antibiofilm | (Kandaswamy et al., 2024) |
| GA-BBR NPs | *S. aureus* | — | 150 μmol/mL | 75 μmol/mL | Antibacterial; Antibiofilm  Non-toxic | (Lu et al., 2023) |
| MPS-NPs | *S. aureus* | CI（n=4） | 0.5-2 | 0.125-0.5 | Antibacterial; Antibiofilm | (Abd El-Hamid et al., 2023) |
| MTN-PEI | *E. coli* | — | + | 1000/5000 | Antibacterial | (Zuo et al., 2024) |
| Nanoparticles of *berberine* and flavonoid glycosides | *S. aureus* | — | 0.075 μmol/mL | 0.05 μmol/mL | Antibacterial; Non-toxic | (Li et al., 2019) |
|  | *S. aureus* | — | 0.075 μmol/mL/ | >0.2 μmol/mL | Antibacterial; Non-toxic |  |
| [PLGA nanoparticles](https://www.mdpi.com/search?q=PLGA+nanoparticles) | [*E. faecalis*](https://www.mdpi.com/search?q=Enterococcus+faecalis) | — | + | + | Antibacterial; Antioxidant | (Marques et al., 2024) |

**Note：AgNPs,** silver nanoparticles; **AuNP-conjugated BER,** berberine was conjugated with gold nanoparticles; **BBC–ZnAl-LDH biohybrids,** delivery system for berberine chloride based on the nanocarrier ZnAl-layered double hydroxide; **BBR-loaded SeNPs,** selenium nanoparticles; **BBR/CGA nanoparticles,** berberine and chlorogenic acid-assembled nanoparticles; **BBR-EGCG NPs,** co-assembled nanocomplexes comprising epigallocatechin gallate and berberine; **BBR-PLGA-LrNs,** lipids Extracted from Mycobacterial Membrane and Enveloped PLGA Nanoparticles for Encapsulating berberine; **BBR-TA NPs,** co-assembled berberine–tannic acid nanoparticles; **BH-CMC hydrogel,** a berberine hydrochloride-carboxymethyl chitosan hydrogel; **BH@MTN-PEI**, graphene oxide-loaded aptamer/berberine bifunctional complex; **BR-loaded NPs,** β-lactoglobulin encapsulate berberine; **BRB-loaded shellac NPs,** berberine based on shellac nanoparticles; **CABs,** using berberine as the photosensitizers core with different length alkyl chains were designed; **CA-BBR NPs,** berberine and cinnamic acid self-assemble into nanoparticles; **Co-amorphous PTM-BCL,** platensimycin-berberine chloride co-amorphous drug system; **Cu @ Bbc,** synergistic berberine chloride and Curcumin-Loaded nanofiber; **GA-BBR NPs,** combination usage of traditional herb medicine, one carrier-free binary nanoparticles; **MPS-NPs,** mesoporous silica nanoparticles; **MTN-PEI,** mesoporous titanium nanoparticles-ethylene imine polymer; **PLGA nanoparticles,** nanoparticle of poly (lactic-co-glycolic acid) loaded with berberine and evaluate.

**References**

Abd El-Hamid, M.I., Ibrahim, D., Elazab, S.T., Gad, W.M., Shalaby, M., El-Neshwy, W.M., et al. (2023). Tackling strong biofilm and multi-virulent vancomycin-resistant Staphylococcus aureus via natural alkaloid-based porous nanoparticles: perspective towards near future eradication. *Front Cell Infect Microbiol* 13**,** 1287426. doi: 10.3389/fcimb.2023.1287426.

Alharthi, S., Popat, A., Ziora, Z.M., and Moyle, P.M. (2023). Sortase A Inhibitor Protein Nanoparticle Formulations Demonstrate Antibacterial Synergy When Combined with Antimicrobial Peptides. *Molecules* 28(5). doi: 10.3390/molecules28052114.

Bhatia, E., Sharma, S., Jadhav, K., and Banerjee, R. (2021). Combinatorial liposomes of berberine and curcumin inhibit biofilm formation and intracellular methicillin resistant Staphylococcus aureus infections and associated inflammation. *J Mater Chem B* 9(3)**,** 864-875. doi: 10.1039/d0tb02036b.

Djebbi, M.A., Elabed, A., Bouaziz, Z., Sadiki, M., Elabed, S., Namour, P., et al. (2016). Delivery system for berberine chloride based on the nanocarrier ZnAl-layered double hydroxide: Physicochemical characterization, release behavior and evaluation of anti-bacterial potential. *International Journal of Pharmaceutics* 515(1-2)**,** 422-430. doi: 10.1016/j.ijpharm.2016.09.089.

Hasan, M.F., Jawad Kadhim, M., and Al-Awady, M.J. (2023). The Impact of Berberine Loaded Selenium Nanoparticles on K. pneumoniae and Candida albicans Antibiotics Resistance Isolates. *Arch Razi Inst* 78(3)**,** 1005-1015. doi: 10.22092/ari.2022.359898.2509.

Huang, X., Wang, P., Li, T., Tian, X., Guo, W., Xu, B., et al. (2020). Self-Assemblies Based on Traditional Medicine Berberine and Cinnamic Acid for Adhesion-Induced Inhibition Multidrug-Resistant Staphylococcus aureus. *ACS Appl Mater Interfaces* 12(1)**,** 227-237. doi: 10.1021/acsami.9b17722.

Kandaswamy, K., Prasad Panda, S., Subramanian, R., Khan, H., Rafi Shaik, M., Althaf Hussain, S., et al. (2024). Synergistic berberine chloride and Curcumin-Loaded nanofiber therapies against Methicillin-Resistant Staphylococcus aureus Infection: Augmented immune and inflammatory responses in zebrafish wound healing. *Int Immunopharmacol* 140**,** 112856. doi: 10.1016/j.intimp.2024.112856.

Li, T., Wang, P.L., Guo, W.B., Huang, X.M., Tian, X.H., Wu, G.R., et al. (2019). Natural Berberine-Based Chinese Herb Medicine Assembled Nanostructures with Modified Antibacterial Application. *Acs Nano* 13(6)**,** 6770-6781. doi: 10.1021/acsnano.9b01346.

Lin, Y.H., Lin, J.H., Chou, S.C., Chang, S.J., Chung, C.C., Chen, Y.S., et al. (2015). Berberine-loaded targeted nanoparticles as specific Helicobacter pylori eradication therapy: in vitro and in vivo study. *Nanomedicine (Lond)* 10(1)**,** 57-71. doi: 10.2217/nnm.14.76.

Lu, J.H., Wang, Z.J., Cai, D.S., Lin, X.Y., Huang, X.M., Yuan, Z.H., et al. (2023). Carrier-Free Binary Self-Assembled Nanomedicines Originated from Traditional Herb Medicine with Multifunction to Accelerate MRSA-Infected Wound Healing by Antibacterial, Anti-Inflammation and Promoting Angiogenesis. *International Journal of Nanomedicine* 18**,** 4885-4906. doi: 10.2147/ijn.S422944.

Marques, C., Grenho, L., Fernandes, M.H., and Costa Lima, S.A. (2024). Improving the Antimicrobial Potency of Berberine for Endodontic Canal Irrigation Using Polymeric Nanoparticles. *Pharmaceutics* 16(6). doi: 10.3390/pharmaceutics16060786.

Ning, Y., Wang, X., Chen, P., Liu, S., Hu, J., Xiao, R., et al. (2022). Targeted inhibition of methicillin-resistant Staphylococcus aureus biofilm formation by a graphene oxide-loaded aptamer/berberine bifunctional complex. *Drug Deliv* 29(1)**,** 1675-1683. doi: 10.1080/10717544.2022.2079768.

Sadeghi, S., Agharazi, F., Hosseinzadeh, S.A., Mashayekhi, M., Saffari, Z., Shafiei, M., et al. (2024). Gold nanoparticle conjugation enhances berberine's antibacterial activity against methicillin-resistant Staphylococcus aureus (MRSA). *Talanta* 268(Pt 1)**,** 125358. doi: 10.1016/j.talanta.2023.125358.

Tahan, M., Zeraatkar, S., Neshani, A., Marouzi, P., Behmadi, M., Alavi, S.J., et al. (2024). Antibacterial Potential of Biosynthesized Silver Nanoparticles Using Berberine Extract Against Multidrug-resistant Acinetobacter baumannii and Pseudomonas aeruginosa. *Indian J Microbiol* 64(1)**,** 125-132. doi: 10.1007/s12088-023-01136-y.

Tong, J., Hou, X., Cui, D., Chen, W., Yao, H., Xiong, B., et al. (2022). A berberine hydrochloride-carboxymethyl chitosan hydrogel protects against Staphylococcus aureus infection in a rat mastitis model. *Carbohydr Polym* 278**,** 118910. doi: 10.1016/j.carbpol.2021.118910.

Wang, Z., Chen, X., Li, D.X., Bai, E.H., Zhang, H.L., Duan, Y.W., et al. (2022). Platensimycin-berberine chloride co-amorphous drug system: Sustained release and prolonged half-life. *European Journal of Pharmaceutics and Biopharmaceutics* 179**,** 126-136. doi: 10.1016/j.ejpb.2022.09.002.

Zheng, L., Zhu, Y., Sun, Y., Xia, S., Duan, S., Yu, B., et al. (2023a). Flexible Modulation of Cellular Activities with Cationic Photosensitizers: Insights of Alkyl Chain Length on Reactive Oxygen Species Antimicrobial Mechanisms. *Adv Mater* 35(35)**,** e2302943. doi: 10.1002/adma.202302943.

Zheng, T.T., Chen, H., Wu, C.Y., Wang, J.R., Cui, M.Y., Ye, H.Y., et al. (2024). Fabrication of Co-Assembly from Berberine and Tannic Acid for Multidrug-Resistant Bacteria Infection Treatment (vol 15, 1782, 2023). *Pharmaceutics* 16(11). doi: 10.3390/pharmaceutics16111418.

Zheng, T.T., Cui, M.Y., Chen, H., Wang, J.R., Ye, H.Y., Zhang, Q.Q., et al. (2023b). Co-assembled nanocomplexes comprising epigallocatechin gallate and berberine for enhanced antibacterial activity against multidrug resistant Staphylococcus aureus. *Biomedicine & Pharmacotherapy* 163. doi: 10.1016/j.biopha.2023.114856.

Zuo, F.J., Wang, B.Y., Wang, L.Z., He, J., and Qiu, X.L. (2024). UV-Triggered Drug Release from Mesoporous Titanium Nanoparticles Loaded with Berberine Hydrochloride: Enhanced Antibacterial Activity. *Molecules* 29(7). doi: 10.3390/molecules29071607.
