# Supplementary material for: Berberine and its nanoformulations and extracts: potential strategies and future perspectives against multi-drug resistant bacterial infections
Source: Front Microbiol. 2025 Sep 2;16:1643409. doi: 10.3389/fmicb.2025.1643409 (PMC12436466; doi:10.3389/fmicb.2025.1643409)
Supplement: Supplementary file 1 [file Data_Sheet_1.zip › Supplementary Table 1.docx]

**Table S1 Antibacterial activity of BBR extracts against bacteria**

| **Antifungal agents** | **Species** | **Identifier** | **MIC/MBC (µg/mL)** | **References** |
| --- | --- | --- | --- | --- |
| Aqueous extracts of *A. mexicana* | *S. aureus* | — | 800000 | (More et al., 2017) |
|  | *B. cereus* | — | 800000 |  |
|  | *E. coli* | — | 800000 |  |
|  | *P. aeruginosa* | — | 800000 |  |
| Methanolic extracts of *A. mexicana* | *S. aureus* | — | 800000 |  |
|  | *B. cereus* | — | 800000 |  |
|  | *E. coli* | — | 800000 |  |
|  | *P. aeruginosa* | — | 800000 |  |
| Aqueous extracts of *Berberis heterophylla* Root | *S. aureus* | ATCC 25923 | 500 -1000 | (Freile et al., 2003) |
|  | *E. faecali* | ATCC 11198 | 500-1000 |  |
|  | *P. aeruginosa* | ATCC 27853 | 500 -1000 |  |
|  | *E. coli* | ATCC 35218 | 500 -1000 |  |
| Aqueous extracts of *Berberis heterophylla* Stem | *S. aureus* | ATCC 25923 | 500 -1000 |  |
|  | *E. faecali* | ATCC 11198 | 500 -1000 |  |
|  | *P. aeruginosa* | ATCC 27853 | 500 -1000 |  |

**Table S1 Continued**

| **Antifungal agents** | **Species** | **Identifier** | **MIC/MBC (µg/mL)** | **References** |
| --- | --- | --- | --- | --- |
| Aqueous extracts of *Berberis heterophylla* Stem | *E. coli* | ATCC 35218 | 500-1000 | (Freile et al., 2003) |
| Aqueous extracts of *Berberis heterophylla* Leaves | *S. aureus* | ATCC 25923 | 500-1000 |  |
|  | *E. faecali* | ATCC 11198 | 500-1000 |  |
|  | *P. aeruginosa* | ATCC 27853 | 500-1000 |  |
|  | *E. coli* | ATCC 35218 | 500-1000 |  |
| Aqueous extract of the dried stem bark of *Berberis aristata* | *S. flexneri* | MTCC 1457 | 300/300 | (Joshi et al., 2011) |
|  | *S. sonnei* | MTCC 2957 | 300/300 |  |
|  | *S. dysenteriae* | LMP 0208U | 250/400 |  |
|  | *S. boydii* | ATCC 8700 | 400/500 |  |
| Alcoholic extract of the dried stem bark of *Berberis aristata* | *S. flexneri* | MTCC 1457 | 250/400 |  |
|  | *S. sonnei* | MTCC 2957 | 500/600 |  |
|  | *S. dysenteriae* | LMP 0208U | 125/400 |  |
|  | *S. boydii* | ATCC 8700 | 500/500 |  |
| Aquoethanolic extract of the stem bark of *Berberis aristata* | *E. coli* | ATCC 25922 | 12.5 | (Thakur et al., 2016) |

**Table S1 Continued**

| **Antifungal agents** | **Species** | **Identifier** | **MIC/MBC (µg/mL)** | **References** |
| --- | --- | --- | --- | --- |
| Aquoethanolic extract of the stem bark of *Berberis aristata* | *E. coli* | ATCC 10798 | 25 | (Thakur et al., 2016) |
|  | *E. coli* | CI (n=1) | 12.5 |  |
| *Banxia Xiexin decoction* | *H. pylori* | 26695 | 512 | (Li et al., 2023) |
|  |  | G27 | 512 |  |
|  |  | NSH57 | 256 |  |
|  |  | BHKS159 | 512 |  |
|  |  | CI (n=14) | 256-512 |  |
| *Berberis aetnensis C. Presl* root extracts | *S. epidermidis* | — | 312 | (Musumeci et al., 2003) |
|  | *S. aureus* | ATCC 29213 | 625 |  |
|  | *B. subtilis* | ATCC 6603 | 625 |  |
|  | *E. faecalis* | ATCC 29212 | 78 |  |
| *Berberis aetnensis C. Presl* leaf extracts | *S. epidermidis* | — | 1250 |  |
|  | *S. aureus* | ATCC 29213 | 78 |  |
|  | *B. subtilis* | ATCC 6603 | 78 |  |
| *Berberis* Decoctum D2 | *S. aureus* | ATCC 29213 | 5000 | (Roser et al., 2016) |

**Table S1 Continued**

| **Antifungal agents** | **Species** | **Identifier** | **MIC/MBC (µg/mL)** | **References** |
| --- | --- | --- | --- | --- |
| *Berberis* Decoctum D2 | *S. aureus* | ATCC 43300 | 5000 | (Roser et al., 2016) |
| *Berberis lycium* Methanolic root extract | *K. pneumoniae* | — | 416.6±72.16 | (Malik et al., 2017) |
|  | *E. coli* | — | 1.7±1.18/2.4±1.18 |  |
|  | *P. aeuroginosa* | — | 458.3±72.16 |  |
|  | *S. aureus* | — | 104.1±18.04/583.3±144.3 |  |
|  | *B. subtilis* | — | 114.5±18.04/333.3±72.2 |  |
| *Berberis microphylla* leaf extract | *S. aureus* | ATCC 25923 | 250/750 | (Manosalva et al., 2016) |
|  | *B. cereus* | ATCC 11778 | 333±118/717±118 |  |
|  | *S. epidermidis* | ATCC 12228 | 125/250 |  |
|  | *B. subtilis* | ATCC 6633 | 333±118/717±118 |  |
| *Berberis microphylla* stem extract | *S. aureus* | ATCC 25923 | 167±50/334±100 |  |
|  | *B. cereus* | ATCC 11778 | 125/250 |  |
|  | *S. epidermidis* | ATCC 12228 | 83±30/167±60 |  |
|  | *B. subtilis* | ATCC 6633 | 250/500 |  |
| *Berberis microphylla* root extract | *S. aureus* | ATCC 25923 | 83±30/167±60 |  |

**Table S1 Continued**

| **Antifungal agents** | **Species** | **Identifier** | **MIC/MBC (µg/mL)** | **References** |
| --- | --- | --- | --- | --- |
| *Berberis microphylla* root extract | *B. cereus* | ATCC 11778 | 125/250 | (Manosalva et al., 2016) |
|  | *S. epidermidis* | ATCC 12228 | 83±30/167±60 |  |
|  | *B. subtilis* | ATCC 6633 | 167±50/334±100 |  |
| *Berberis microphylla* stem extract | *S. mutans* | PTCC 1683 | 170.66 ± 73.90 l/256 | (Kazemipoor et al., 2021) |
|  | *S. sobrinus* | PTCC 1601 | 64/128 |  |
|  | *S. sanguinis* | PTCC 1449 | 85.33 ± 36.95/128 |  |
|  | *S.salivaris* | PTCC 1448 | 64/128 |  |
|  | *L. rhamnosus* | PTCC 1637 | 128 /128 |  |
| *Berberis microphylla* Fruit extract | *S. mutans* | PTCC 1683 | 64/128 |  |
|  | *S. sobrinus* | PTCC 1601 | 128/128 |  |
|  | *S. sanguinis* | PTCC 1449 | 64/128 |  |
|  | *S. salivaris* | PTCC 1448 | 64/128 |  |
|  | *L. rhamnosus* | PTCC 1637 | >256/>256 |  |
| *Berberis microphylla* Leaf extract | *S. mutans* | PTCC 1683 | >256/256 |  |

**Table S1 Continued**

| **Antifungal agents** | **Species** | **Identifier** | **MIC/MBC (µg/mL)** | **References** |
| --- | --- | --- | --- | --- |
| *Berberis microphylla* Leaf extract | *S. sobrinus* | PTCC 1601 | >256/>256 | (Kazemipoor et al., 2021) |
|  | *S. sanguinis* | PTCC 1449 | >256/>256 |  |
|  | *S. salivaris* | PTCC 1448 | 64/128 |  |
|  | *L. rhamnosus* | PTCC 1637 | >256/>256 |  |
| CHM *Coptis chinensis* extract | *M. abscessus* | — | 1500/6000 | (Tseng et al., 2020) |
| *Coptis chinensis* extract | *P. acnes* | KCTC3320 | 0.5-1 | (Wijaya et al., 2022) |
| *Coptis chinensis* extract | *M. abscessus* | — | 1500/6000 | (Tseng et al., 2020) |
| *Coptis chinensis* roots extract | *P. acnes* | 3320 | 0.5 | (Lee et al., 2018) |
|  | *S. aureus* | — | 1 |  |
|  | *L. monocytogenes* | — | 1 |  |
| *Corydalis* Tuber extract | *MRSA* | ATCC 33593 | 512000 | (Seo et al., 2024) |
| *Coptidis rhizome* extract | *S. ftyphimurium* | ATCC 6994 | 12500 | (Chang et al., 2014) |
| *Hydrastis canadensis L.* extract | *S. aureus* | ATCC 25923 | 120 | (Scazzocchio et al., 2001) |
|  | *S. aureus* | ATCC 6538P | 31 |  |
|  | *S. sanguinis* | ATCC10556 | 500 |  |

**Table S1 Continued**

| **Antifungal agents** | **Species** | **Identifier** | **MIC/MBC (µg/mL)** | **References** |
| --- | --- | --- | --- | --- |
| *Hydrastis canadensis L.* extract | *E. coli* | ATCC25922 | >1000 | (Scazzocchio et al., 2001) |
|  | *P. aeruginosa* | ATCC27853 | >1000 |  |
| *Mahonia aquifolium* crude extract | *S. epidermidis* | — | 100–>500 | (Slobodníková et al., 2004) |
|  | *S. hominis* | — | 250–>500 |  |
|  | *S. warneri* | — | 100 |  |
|  | *S. lentus* | — | 100 |  |
|  | *S. hyicus* | — | 100 |  |
|  | *P. acnes* | — | 25–50 |  |
| *Rhizoma coptidis* extract | *MRSA* | ATCC 43300 | 8 | (Luo et al., 2014) |

**Note: *B. cereus*,** *Bacillus cereus*; ***L. rhamnosus***, *Lactobacillus rhamnosus*; ***S. hyicus***, *Staphylococcus hyicus*; ***S. salivaris***, *Streptococcus salivarius*; ***S. sobrinus***, *Streptococcus sobrinus;*

**References**

Chang, C.H., Yu, B., Su, C.H., Chen, D.S., Hou, Y.C., Chen, Y.S., et al. (2014). Coptidis rhizome and Si Jun Zi Tang can prevent Salmonella enterica serovar Typhimurium infection in mice. *PLoS One* 9(8)**,** e105362. doi: 10.1371/journal.pone.0105362.

Freile, M.L., Giannini, F., Pucci, G., Sturniolo, A., Rodero, L., Pucci, O., et al. (2003). Antimicrobial activity of aqueous extracts and of berberine isolated from Berberis heterophylla. *Fitoterapia* 74(7-8)**,** 702-705. doi: 10.1016/s0367-326x(03)00156-4.

Joshi, P.V., Shirkhedkar, A.A., Prakash, K., and Maheshwari, V.L. (2011). Antidiarrheal activity, chemical and toxicity profile of Berberis aristata. *Pharm Biol* 49(1)**,** 94-100. doi: 10.3109/13880209.2010.500295.

Kazemipoor, M., Fadaei Tehrani, P., Zandi, H., and Golvardi Yazdi, R. (2021). Chemical composition and antibacterial activity of Berberis vulgaris (barberry) against bacteria associated with caries. *Clin Exp Dent Res* 7(4)**,** 601-608. doi: 10.1002/cre2.379.

Lee, J.W., Kang, Y.J., Choi, H.K., and Yoon, Y.G. (2018). Fractionated Coptis chinensis Extract and Its Bioactive Component Suppress Propionibacterium acnes-Stimulated Inflammation in Human Keratinocytes. *J Microbiol Biotechnol* 28(6)**,** 839-848. doi: 10.4014/jmb.1712.12051.

Li, X.H., Xu, J.Y., Wang, X., Liao, L.J., Huang, L., Huang, Y.Q., et al. (2023). BanXiaXieXin decoction treating gastritis mice with drug-resistant Helicobacter pylori and its mechanism. *World J Gastroenterol* 29(18)**,** 2818-2835. doi: 10.3748/wjg.v29.i18.2818.

Luo, J.Y., Yan, D., and Yang, M.H. (2014). Study of the anti-MRSA activity of Rhizoma coptidis by chemical fingerprinting and broth microdilution methods. *Chin J Nat Med* 12(5)**,** 393-400. doi: 10.1016/s1875-5364(14)60049-2.

Malik, T.A., Kamili, A.N., Chishti, M.Z., Ahad, S., Tantry, M.A., Hussain, P.R., et al. (2017). Breaking the resistance of Escherichia coli: Antimicrobial activity of Berberis lycium Royle. *Microb Pathog* 102**,** 12-20. doi: 10.1016/j.micpath.2016.11.011.

Manosalva, L., Mutis, A., Urzúa, A., Fajardo, V., and Quiroz, A. (2016). Antibacterial Activity of Alkaloid Fractions from Berberis microphylla G. Forst and Study of Synergism with Ampicillin and Cephalothin. *Molecules* 21(1)**,** 76. doi: 10.3390/molecules21010076.

More, N.V., Kharat, K.R., and Kharat, A.S. (2017). Berberine from Argemone mexicana L exhibits a broadspectrum antibacterial activity. *Acta Biochim Pol* 64(4)**,** 653-660. doi: 10.18388/abp.2017_1621.

Musumeci, R., Speciale, A., Costanzo, R., Annino, A., Ragusa, S., Rapisarda, A., et al. (2003). Berberis aetnensis C. Presl. extracts: antimicrobial properties and interaction with ciprofloxacin. *Int J Antimicrob Agents* 22(1)**,** 48-53. doi: 10.1016/s0924-8579(03)00085-2.

Roser, E., Gründemann, C., Engels, I., and Huber, R. (2016). Antibacterial in vitro effects of preparations from Anthroposophical Medicine. *BMC Complement Altern Med* 16(1)**,** 372. doi: 10.1186/s12906-016-1350-3.

Scazzocchio, F., Cometa, M.F., Tomassini, L., and Palmery, M. (2001). Antibacterial activity of Hydrastis canadensis extract and its major isolated alkaloids. *Planta Med* 67(6)**,** 561-564. doi: 10.1055/s-2001-16493.

Seo, Y., Kim, M., and Kim, T.J. (2024). Enhanced Efficacy of Ciprofloxacin and Tobramycin against Staphylococcus aureus When Combined with Corydalis Tuber and Berberine through Efflux Pump Inhibition. *Antibiotics (Basel)* 13(5). doi: 10.3390/antibiotics13050469.

Slobodníková, L., Kost'álová, D., Labudová, D., Kotulová, D., and Kettmann, V. (2004). Antimicrobial activity of Mahonia aquifolium crude extract and its major isolated alkaloids. *Phytother Res* 18(8)**,** 674-676. doi: 10.1002/ptr.1517.

Thakur, P., Chawla, R., Goel, R., Narula, A., Arora, R., and Sharma, R.K. (2016). Augmenting the potency of third-line antibiotics with Berberis aristata: In vitro synergistic activity against carbapenem-resistant Escherichia coli. *J Glob Antimicrob Resist* 6**,** 10-16. doi: 10.1016/j.jgar.2016.01.015.

Tseng, C.Y., Sun, M.F., Li, T.C., and Lin, C.T. (2020). Effect of Coptis chinensis on Biofilm Formation and Antibiotic Susceptibility in Mycobacterium abscessus. *Evid Based Complement Alternat Med* 2020**,** 9754357. doi: 10.1155/2020/9754357.

Wijaya, V., Janďourek, O., Křoustková, J., Hradiská-Breiterová, K., Korábečný, J., Sobolová, K., et al. (2022). Alkaloids of Dicranostigma franchetianum (Papaveraceae) and Berberine Derivatives as a New Class of Antimycobacterial Agents. *Biomolecules* 12(6). doi: 10.3390/biom12060844.
